# A comprehensive evaluation of adverse childhood experiences, social–emotional impairments, and neurodevelopmental disorders in cannabis-use disorder: Implications for clinical practice

**DOI:** 10.1192/j.eurpsy.2023.2436

**Published:** 2023-09-13

**Authors:** Giada Trovini, Emanuela Amici, Piergiorgio Bauco, Marta Matrone, Ginevra Lombardozzi, Valeria Giovanetti, Georgios D. Kotzalidis, Sergio De Filippis

**Affiliations:** 1 Clinica Villa Von Siebenthal, Rome, Italy; 2 Department of Psychiatry, Università Politecnica delle Marche, Ancona, Italy; 3 NESMOS Department, Sapienza University of Rome, Faculty of Medicine and Psychology, Rome, Italy

**Keywords:** adverse childhood experiences, cannabis, history of pre-onset conditions, mental disorders, substance-use disorders

## Abstract

**Background:**

Adverse childhood experiences (ACEs), social–emotional impairments (SEIs), and neurodevelopmental disorders (NDs) are frequent in psychiatric disorders, including substance-use disorders. We aimed to determine the prevalence of ACE, SEI, or ND in individuals with cannabis-use disorder (CUD). We compared individuals with preCUD-onset ACE, SEI, or ND to those without.

**Methods:**

We crosssectionally studied 323 inpatients or outpatients with a history of past or current CUD, aged 12–35 years (mean age 22.94 ± 4.79), 64.5% of whom were male. The sample was divided into two groups: the non-premorbid (N = 52) and the premorbid ACE/SEI/ND group (N = 271). Within the premorbid group, further subgroups were based on ACEs, SEI, and NDs. We also analyzed other substance use and psychiatric symptoms/diagnoses based on the non-premorbid-premorbid dichotomy in the CUD sample.

**Results:**

Pre-CUD ACE-SEI-ND had higher prevalence of bipolar, schizoaffective, borderline personality, and attention-deficit/hyperactivity disorders, and a history of agitation, hallucinations, and self-injury. The ACE group had higher rates of agitation, depression, delusions, hallucinations, eating disorders, and use of cocaine, amphetamines, and hallucinogens than the SEI or ND. Patients in the premorbid group initiated cannabis use at an earlier age, experienced the first comorbid psychiatric episode earlier, and were hospitalized earlier than those in the non- premorbid ACE-SEI-ND group.

**Conclusions:**

PreCUD-onset ACE, SEI, or ND conditions in individuals with CUDare linked to earlier onset of comorbid mental illness. Furthermore, ACEs contribute to significant and potentially severe clinical symptoms, as well as the use of substances other than cannabis.

## Introduction

Substance-use disorders (SUDs) are a significant global health and social issue, often leading to major psychiatric disorders such as schizophrenia, bipolar disorder, and depressive disorders [1]. The experiences individuals have during childhood and adolescence can have a long-lasting impact on their overall health and well-being throughout their lives [2], particularly in relation to future illicit drug use [3, 4]. The long-term negative effects of premorbid experiences and events on mental health have been extensively studied [5, 6].

Patients with SUDs frequently have a higher prevalence of adverse childhood experiences (ACEs) [7–11]. ACEs, including emotional and physical abuse, neglect during childhood, and other traumatic experiences, act as risk factors for SUDs. ACEs consistently contribute to an increased risk of substance use, including early initiation of substance use and subsequent development of SUDs [12–17].

Attention-deficit/hyperactivity disorder (ADHD) is the most common psychiatric disorder in childhood, with a worldwide pooled prevalence of 5.3% [18, 19]. Cross-sectional epidemiological studies have found an association between ADHD and drug use, including SUDs [20, 21].

Several studies show that ADHD and SUD are mutually interconnected disorders. Approximately 15% of young adults with ADHD have a comorbid SUD [20]. In line with existing ADHD literature, individuals with ADHD frequently use substances such as tetrahydrocannabinol (THC), central nervous system stimulants, alcohol, benzodiazepines, and opioids [22]. Although previous research has explored the connection between premorbid conditions and substance use and there is growing interest in understanding the mechanisms linking preCUD-onset conditions/functioning with cannabis use [23–26], there is limited knowledge about how different patterns of premorbid conditions influence cannabis use and mental health. Existing studies primarily focus on psychoses and schizophrenia spectrum disorders [27–30]. This study aimed to investigate the impact of early adverse experiences and preCUD-onset conditions on the onset, clinical presentation, and symptoms of young adults. Specifically, we aimed to assess the presence of CUD-onset conditions in patients with past or current history of cannabis use in a real-world cannabis-use disorder (CUD) cohort observed longitudinally, comparing groups based on their preCUD-onset status and focusing on the occurrence of ACEs, SEI or NDs. We hypothesized that preCUD-onset ACEs, SEI or ND would be more prevalent in patients with CUD and anticipated that this subgroup would exhibit more severe comorbid psychiatric symptoms.

## Materials and methods

### Study design

This cross-sectional, retrospective, real-world study was conducted between June 2021 and December 2022 at the inpatient and outpatient units of Von Siebenthal Hospital in Rome. The study aimed to determine the presence of preCUD-onset conditions in patients with a history of or current cannabis use. Trained psychiatrists conducted interviews with patients either in person or *via* telephone, and medical records were reviewed. To classify preCUD-onset conditions, we referred to Felitti et al.’s classification [12] and Goddard’s ACE Pyramid frame [31], which grouped the sample into three macro-areas:

- ACEs, including psychological, physical, or sexual abuse; physical and emotional neglect; other traumatic childhood experiences; exposure to household violence; or living with mentally ill family members or those with SUD.
- Social and emotional impairment (SEI), encompassing impairment in social and emotional functioning, difficult economic conditions, poor housing conditions, migration, adoption, anxiety, peer rejection, and limited access to healthcare and education.
- Neurodevelopmental disorders (NDs), based on the DSM-5 classification, which includes intellectual disability (ID), autism spectrum disorder (ASD), attention-deficit/hyperactivity disorder (ADHD), communication disorders, specific learning disorders, movement disorders, and Tic disorders.

Following instructions on how to calculate it, included patients were asked to report the amount of THC in grams per day and any concurrent use of other substances such as alcohol, cocaine, opioids, amphetamines, hallucinogens, and benzodiazepines. Patients were asked “How much cannabis have you used before seeking emergency medical treatment following cannabis use?” (to report in mg) [32]; if patients could not relate the amount to mg, we asked for the number of joints used and multiplied them per 0.32, according to the latest indications [33]. Each patient, when hospitalized, was assigned to a multiprofessional team (psychiatrist, psychologist, psychiatric rehabilitation technician, and nurses). The clinical evaluation was conducted with the patient and at least one family member/caregiver when the patient was not at legal age. At the end of hospitalization, patients were followed up with check-ups and monitoring visits, in person and also through telephone calls.

### Sample

Out of the 1000 consecutive patients interviewed, 323 met the eligibility criteria and were included in the study. Inclusion criteria were patients with a previous or current CUD. Exclusion criteria were patients with a current diagnosis of moderate to severe Intellectual Disability, those with serious medical conditions, and pregnant women or those planning pregnancy. Among the included patients, 209 (64.5%) were males and 114 (35.5%) were females, with a mean age of 22.94 years (SD = 4.79), ranging from 12 to 35 years. 10% of the entire sample were under the age of 18 years (Table 2). The sample was then divided into two groups: the preCUD-onset group, including those with a preCUD-onset ACE, SEI, or ND condition in their clinical history, and another group which included those without any of these preCUD-onset conditions.

### Assessments

We collected patients’ main sociodemographic characteristics using a specifically designed questionnaire. Clinical interviews were conducted either in person or via telephone for all patients. Psychiatric diagnoses were made based on the DSM-5 criteria using the Structured Clinical Interview for DSM-5-Clinician Version (SCID-5-CV) [34]. ND diagnoses were determined by our Child Neuropsychiatry Centre, following the DSM-5 criteria.

Our focus was on identifying predictors of clinical status (symptoms) based on preCUD-onset conditions (ACEs, SEI, or ND) and whether these were related to the age of onset of CUD and the dose/intensity of cannabis use. We also compared the non-preCUD-onset group with the preCUD-onset group (and its subgroups) in terms of age at cannabis initiation, age at clinical onset, age at first hospitalization, symptoms, diagnosis, and use of other substances. Additionally, we sought to explore possible differences among the three preCUD-onset macro-areas (ACEs, SEI, and NDs).

### Ethics

Patients provided written informed consent before participating in any study procedures. For underage participants, information regarding the study procedures and objectives was provided also to their parents and/or legal guardians/tutors. The consent form and experimental procedures were approved by the ethics committee of Rome2 Health Authority (study 331-306-00387), in accordance with internationally accepted criteria for ethical research. The study was conducted in compliance with the Principles of Human Rights, as adopted by the World Medical Association at the 18th WMA General Assembly, held in Helsinki, Finland, in June 1964, and subsequently amended at the 64th WMA General Assembly, held in Fortaleza, Brazil, in October 2013.

### Data analysis

Comparative analysis of demographic, clinical, and symptomatic characteristics, diagnosis, and type of substance use among different subgroups was performed using Student’s *t*-test for continuous variables and chi-square test for categorical variables (Fisher exact-test when appropriate). Considering the number of subjects and the confirmatory nature of our study, we conservatively used two-tailed significance levels with a threshold of *p* < 0.05. All analyses were conducted using the SPSS statistical package (version 25.0), IBM Corporation, Armonk, New York, 2016.

## Results

### Patients’ characteristics

The sociodemographic and clinical characteristics of the patients are presented in Table 1. The mean age of the total sample was 22.9 years (SD = 4.7). The average age at which cannabis use started was 14.4 years (SD = 2.2), while the average age at which clinical symptoms of psychiatric diseases emerged was 16.3 years (SD = 3.04). Thus, the onset of cannabis use preceded the onset of clinical symptoms by approximately 2 years. The duration of untreated psychosis (DUP) was 1.3 years, and the mean age of the first hospitalization was 17.6 years (SD = 3.6).

**Table 1.** Patients’ sociodemographic characteristics and clinically relevant variables for SUDs (N = 323)

Continuous variables	Mean (SD)
Age	22.94 (4.69)
Age at onset of psychopathology	16.32 (3.04)
Age at onset of cannabis use	14.39 (2.20)
Interval between cannabis use and psychopathological onset	1.98 (2.03)
Age at first hospitalization	17.59 (3.57)

Nominal variables	N (%)
Sex	
Male	209 (64.7)
Female	114 (35.5)
Patients aged less than 18 years	35 (10.8)
Daily THC dose	
<1 g/day	114 (35.3)
1–2 g/day	119 (36.8)
2–4 g/day	89 (27.6)
>4 g/day	1 (0.03)

Type of substance-use disorder	N (%)
Polysubstance use	205 (63.5)
Cocaine	134 (41.5)
Opioids	24 (7.4)
Amphetamines	49 (15.2)
Hallucinogens	32 (9.9)
Alcohol	117 (36.2)
Benzodiazepines	24 (7.4)

Abbreviations: SD, standard deviation; SUDs, substance-use disorders; THC, Delta^9^-tetrahydrocannabinol.

### Main findings

All patients included in the study had a history of THC use either at the time of evaluation or during their lifetime. The sample was divided into two groups: one group with a preCUD-onset condition in their clinical history, and the other group without any preCUD-onset condition (Table 2). In the preCUD-onset group, the average age of cannabis use onset was 14.1 years (SD = 2.0), while in the non-preCUD-onset group, it was 15.9 years (SD = 2.6). The average age of symptom onset and first hospitalization in patients with a preCUD-onset condition were 15.9 years (SD = 2.9) and 17.0 years (SD = 3.1), respectively. In contrast, in the group without any preCUD-onset condition, the respective ages were 18.4 years (SD = 3.2) and 20.4 years (SD = 4.6), showing a difference of approximately 3 years between the two groups. These differences were statistically significant.

**Table 2.** Sociodemographic characteristics of the sample and clinically relevant variables in the preCUD-onset conditions *versus* nonpreCUD-onset conditions groups (N = 323)

Variables, Mean (SD)	PreCUD-onset conditions (N = 271)	NonpreCUD-onset conditions (N = 52)	F		p	
Age at onset of cannabis use	14.10 (1.99)	15.94 (2.62)	**33.76**		**<0.001**	
Interval between cannabis use and psychopathology	1.89 (1.96)	2.42 (2.03)	3.04		0.082	
Age at onset of psychopathology	15.93 (2.86)	18.37 (3.18)	**30.70**		**<0.001**	
Age at first hospitalization	17.05 (3.08)	20.37 (4.60)	**42.33**		**<0.001**	

N (%)			*χ*^2^			*p*
Sex						
Male	171 (81.8)	38 (18.2)				
Female	100 (87.7)	14 (12.3)	1.90			0.110
Diagnosis N (%)						
Schizophrenia	31 (93.9)	2 (6.1)	2.74			0.071
Bipolar disorder	116 (93.5)	8 (6.5)	**13.87**			**0.000**
Depressive disorders	36 (81.8)	8 (18.2)	0.16			0.413
Schizoaffective disorder	88 (72.1)	34 (27.9)	**20.10**			**0.000**
Borderline personality disorder	57 (96.6)	2 (3.4)	**8.63**			**0.001**
Attention-deficit/hyperactivity disorder	55 (100)	0 (0.0)	**37.31**			**0.000**
Antisocial personality disorder	3 (100)	0 (0.0)	0.58			0.590
Mild intellectual disability	2 (100)	0 (0.0)	0.38			0.714
Schizoid personality disorder	1 (100)	0 (0.0)	0.19			0.839
Avoidant personality disorder	1 (100)	0 (0.0)	0.19			0.839
Autism spectrum disorder	1 (100)	0 (0.0)	0.19			0.839
Daily THC dose		0 (0.0)				
<1 g/day	98 (86.0)	16 (14.0)	3.84			0.279
1–2 g/day	103 (86.6)	16 (13.4)				
2–4 g/day	69 (77.5)	20 (22.5)				
>4 g/day	1 (100)	0 (0.0)				
Substance use						
Cocaine	100 (74.6)	34 (25.4)	**14.58**			**0.000**
Opioids	19 (79.2)	5 (20.8)	0.43			0.339
Amphetamines	33 (83.9)	16 (16.1)	**11.62**			**0.001**
Hallucinogens	19 (59.4)	13 (40.6)	**15.81**			**0.000**
Alcohol	98 (83.8)	19 (16.2)	0.003			0.538
Benzodiazepines	17 (70.8)	7 (29.2)	3.27			0.071

Symptoms, N (%)						
Agitation	119 (91.5)	11 (8.5)		**9.39**	**0.001**	
Dyscontrol	65 (89.0)	8 (11.0)		1.84	0.117	
Withdrawal	64 (84.2)	12 (15.8)		0.007	0.546	
Mood depression	64 (78.0)	18 (22.0)		2.78	0.070	
Delusions	84 (79.2)	22 (20.8)		2.53	0.078	
Hallucinations	54 (74.0)	19 (26.0)		**6.88**	**0.009**	
Panic	47 (82.5)	10 (17.5)		0.10	0.438	
Self-harm	84 (92.3)	7 (7.7)		**6.62**	**0.006**	
Eating disorder	16 (100)	0 (0.0)		3.23	0.056	

Note: Significant results in bold characters.

Abbreviations: F, between/within sample variation, a coefficient of Analysis of Variance (ANOVA); *p*, statistical probability (significance); SD, standard deviation; THC, Delta^9^-tetrahydrocannabinol; *χ*^2^, chi-squared test.

The DSM-5 diagnoses are presented in Table 2. Bipolar disorder (N = 116 *versus* 8, *χ*^2^ = 13.87, *p* < 0.0001), schizoaffective disorder (N = 88 *versus* 34, *χ*^2^ = 20.10, *p* < 0.0001), ADHD (N = 55 *versus* 0, *χ*^2^ = 37.31, *p* < 0.0001), and borderline personality disorder (BPD) (N = 57 *versus* 2, *χ*^2^ = 8.63, *p* = 0.001) were more prevalent in the preCUD-onset group compared to the non-preCUD-onset group. There were no statistically significant differences in the other diagnoses.

We also examined the different symptoms at clinical onset. “Agitation” (N = 119 *versus* 11, *χ*^2^ = 9.39, *p* = 0.001), “Hallucinations” (N = 54 *versus* 19, *χ*^2^ = 6.88, *p* = 0.009), and “Self-harm” (N = 84 *versus* 7, *χ*^2^ = 6.62, *p* = 0.006) were more frequent in the preCUD-onset group compared to the non-preCUD-onset group.

### Clinical characteristics of different patient subgroups

Tables 3 and 4 presents a comparison between the non-preCUD-onset (NO) group and the preCUD-onset groups (ACE, SEI, or ND) in terms of symptoms at clinical onset (Table 3) and the history of substance use (Table 4). The ACE group had a higher prevalence of “Agitation” (N = 61 for ACE versus 30 for ND *versus* 27 for SEI, *χ*^2^ = 10.14, *p* = 0.017), “Depression” (N = 46 for ACE *versus* 9 for ND *versus* 9 for SEI, *χ*^2^ = 12.31, *p* = 0.006), and “Eating disorder” (N = 12 for ACE *versus* 3 for ND *versus* 1 for SEI, *χ*^2^ = 7.59, *p* = 0.055) compared to the other groups. “Delusions” (N = 33 for ACE and N = 31 for SEI *versus* 20 for ND, *χ*^2^ = 15.58, *p* = 0.001) and “Hallucinations” (N = 21 for ACE and SEI *versus* 12 for ND, *χ*^2^ = 14.75, *p* = 0.002) were more prevalent in the ACE and SEI groups and less prevalent in the ND group.

**Table 3.** Symptomatology at clinical onset as recollected by each patient (N = 323) according to nonpreCUD-onset condition/preCUD-onset condition group subtype

Symptoms, N (%)	NO (N = 53)	ACE (N = 147)	SEI (N = 65)	ND (N = 58)	χ^2^	p
Agitation	12 (9.2)	61 (46.9)	27 (20.8)	30 (23.1)	10.14	**0.017**
Impulse dyscontrol	9 (12.3)	34 (46.6)	12 (16.4)	18 (24.7)	3.97	0.264
Withdrawal	12 (15.8)	33 (43.3)	16 (21.1)	15 (19.7)	0.337	0.953
Mood depression	18 (22.0)	46 (56.1)	9 (11.0)	9 (11.0)	12.31	**0.006**
Delusions	22 (20.8)	33 (31.1)	31 (29.2)	20 (18.9)	15.58	**0.001**
Hallucinations	19 (26.0)	21 (28.8)	21 (28.8)	12 (16.4)	14.75	**0.002**
Panic	10 (17.5)	30 (52.6)	11 (19.3)	6 (10.5)	2.97	0.395
Self-harm	7 (7.7)	44 (48.4)	22 (24.2)	18 (19.8)	7.35	0.061
Eating disorder	0 (0)	12 (75.0)	1 (6.3)	3 (18.8)	7.59	0.**055**

Note: Significant results in bold characters.

Abbreviations: ACE, adverse childhood experiences; ND, neurodevelopmental disorders as defined by the DSM-5; NO, no preCUD-onset condition status; *p*, statistical probability (significance); SEI, social and emotional impairment; *χ*^2^, chi-squared test.

**Table 4.** Type of substance use according to nonpreCUD-onset condition/preCUD-onset condition subtype (N = 323)

Substances, N (%)	NO (N = 53)	ACE (N = 147)	SEI (N = 65)	ND (N = 58)	*χ*^2^	p
Cocaine	35 (26.1)	48 (35.8)	28 (20.9)	23 (17.2)	18.03	**0.000**
Opioids	5 (20.8)	10 (41.7)	6 (25.0)	3 (12.5)	1.13	0.770
Amphetamines	16 (32.7)	15 (30.6)	11 (22.4)	7 (14.3)	12.56	**0.006**
Hallucinogens	13 (40.6)	12 (37.5)	3 (9.4)	4 (12.5)	15.82	**0.001**
Alcohol	19 (16.2)	52 (44.4)	23 (19.7)	23 (19.7)	0.36	0.947
Benzodiazepines	7 (29.2)	11 (45.8)	1 (4.2)	5 (20.8)	5.97	0.113

Significant results in bold characters.

Abbreviations: ACE, adverse childhood experiences; ND, neurodevelopmental disorders as defined by the DSM-5; NO, no preCUD-onset condition status; *p*, statistical probability (significance); SEI, social and emotional impairment; *χ*^2^, chi-squared test.

Table 4 reports the type of substance use according to each preCUD-onset condition and the NO group. Specifically, 35.8% of cocaine users had a history of ACE (*versus* 20.9% of SEI and 17.2% of ND, *χ*^2^ = 18.03, *p* < 0.0001). Similarly, 30.6% of amphetamine users had a history of ACE (*versus* 22.4% of SEI and 14.3% of ND, *χ*^2^ = 12.56, *p* = 0.006), and 37.5% of hallucinogen users had a history of ACE (*versus* 9.4% of SEI and 12.5% of ND, *χ*^2^ = 15.82, *p* = 0.001).

ADHD comorbidity was observed in 6 out of 33 patients with schizophrenia (18.2%), 28 out of 124 patients with bipolar disorder (22.6%), 4 out of 44 patients with major depressive disorder (9.1%), 20 out of 122 patients with schizoaffective disorder (16.4%), and 6 out of 59 patients with BPD (10.2%). However, the differences between the groups were not statistically significant (*χ*^2^ = 6.7647; *p* = 0.149, n.s.).

## Discussion

Our findings revealed a correlation between preCUD-onset conditions and early cannabis use, cocaine use, and earlier hospitalization. Furthermore, a connection was observed between preCUD-onset conditions and a predominantly affective dimension of the disorder. These results confirm that preCUD-onset conditions can act as risk factors for SUDs.

Numerous studies have demonstrated the long-term negative effects of preCUD-onset experiences and events on physical and mental health. The sociodemographic and socioeconomic characteristics [35–37], psychiatric history, traumatic events, suicide attempts, and history of substance use affect different outcomes [36], and appear to differ in diagnostic category, illness course and response to treatment. Results support our hypothesis that preCUD-onset conditions represent a vulnerability that is configured early as a risk factor for the initiation of cannabis use, for an earlier onset of clinical symptoms, and for earlier hospitalization. ACEs have both short- and long-term impacts on individual development. Symptoms like aggressiveness, emotional dysregulation, anxiety, and emotional detachment are a possible response to trauma [31]. Childhood trauma is often neglected in diagnosing depression, anxiety, or ADHD and may lead to illness and disability [12, 38]. Childhood trauma was reported in two-thirds of former pediatric psychiatric service referrals, with significantly higher rates of dysfunctional families, neglect, and abuse. Furthermore, these young inpatients suffered from “psychosomatic” or personality disorders and showed comorbidities and impaired functioning [39].

Current prevalence estimates for ASD and ADHD in adulthood are 0.6% [20] and 4.4% [40], respectively. Both disorders have an important impact on development, beginning at an early age. In addition, these disorders can present with co-morbid conditions like SUD. Levels of disability are more severe in patients with ASD or ADHD with co-morbid SUD [41]. We could not confirm a higher prevalence of SUD in patients with an ASD or ADHD diagnosis, as we pooled these two disorders along with other disorders in the ND group. Furthermore, splitting out this group from the one with ACEs, resulted in ND showing less agitation, depression, delusions, hallucinations (hallucinations), cocaine, amphetamine, and hallucinogen use than the ACE group.

Our results support the hypothesis that the use of substances is an attempt at self-treatment to alleviate the suffering of traumatic experiences, symptoms such as anxiety, emotional dysregulation, emotional and social difficulties. Consistently Khantzian et al. [42] speculated that the use of drugs was a way to suppress ADHD symptoms, following the self-medication hypothesis. Individuals with ASD frequently have psychiatric comorbidities, such as social or generalized anxiety disorder and depression. These comorbidities can more and more increase substance risk as a coping strategy. Substance use can reduce anxiety, feelings of inadequacy, and low self-esteem, which accompany and limit social drive [43, 44].

Numerous hypotheses have been advanced to explain the association between SUD and ADHD. The prevalence of SUD seems higher in the parents of ADHD children. Following a developmental perspective, ADHD symptoms usually appear during childhood and adolescence, suggesting a role of this illness as a risk factor for SUD [22]. According to the self-medication hypothesis, patients report the use of drugs as a way to suppress ADHD symptoms [42]. We could not test this due to insufficient subsamples for conducting reliable statistics.

Usually SUDs in adulthood are preceded by affective conditions, such as depressive or anxiety disorders [45]. Patients who experience comorbid depressive and anxiety disorders tend to suffer greater emotional or affective distress and experience more severe substance use symptoms [46]. This is consistent with the self-medication hypothesis.

Depressive symptoms and conduct problems are among the most common symptoms during adolescence and are associated with a high likelihood of polysubstance use [47]. Lifetime prevalence of major depressive disorder and conduct disorder in adolescents was approximately 11.7 and 7.6%, respectively [48]. Adolescents with severe symptoms of depression and conduct problems had the highest probability of using alcohol, cigarettes, and marijuana together [47].

Comparing the diagnoses of the preCUD-onset *versus* non-preCUD-onset groups, we found 93% of patients with bipolar disorder to have a preCUD-onset condition in their history; this was 96% in cases with BPD, both with statistical significance. Our results seem to confirm a link between the presence of a preCUD-onset condition and disorders with a strong affective component (Bipolar Disorder, Schizoaffective Disorder, ADHD, and BPD). It is interesting that we had 28 patients with ADHD who were comorbid with Bipolar Disorder, i.e., 24.14% of the Bipolar Disorder sample, while these comorbid patients represented 50.91% of the total ADHD sample. A recent meta-analysis of more than 600,000 patients in 18 countries found an average of 17.11% of patients with BD to have ADHD, while those with ADHD were comorbid for Bipolar Disorder for about 8% [47]; the differences from our study regarded not so much how many patients with bipolar disorder had ADHD, but rather how many patients with ADHD had bipolar disorder. However, this meta-analysis found a considerable heterogeneity between studies that depended on diagnostic system used, sample size, and geographical location, a fact that could account for the differences found [49].

Childhood trauma is an important risk factor for development of mood disorders [50, 51]. A cohort study of over 11 million adults found that those who disclosed ACE were 2.14 times more likely to have a psychiatric diagnosis, most prominently mood disorders [52]. Child maltreatment worsens the probability of developing bipolar spectrum disorder [53] and is associated to a worse clinical picture [54] and outcome [55]. Bipolar disorder and SUD often coexist and this is also true when bipolar disorder is associated with ADHD [56].

Our data show the presence of polysubstance use in patients of our sample. In particular, the use of cocaine, hallucinogens, and amphetamines is more frequent in patients with preCUD-onset conditions than in the non-preCUD-onset group.

Subsequently we examined symptoms at clinical onset. The comparison showed that “Agitation”, “Hallucinations”, and “Self-harm” are significantly more represented in the group with preCUD-onset conditions. This result seems to be consistent with what has been stated above regarding the results of age at psychotic onset and age at first hospitalization. If it is true that the preCUD-onset conditions predispose to the earlier development of symptoms requiring hospitalization, it is not surprising that among the most represented symptoms in the group there are psychomotor agitation and self-harming behaviors.

Exposure to adversity events early in life has been associated with many negative consequences, including mental health problems, substance use, social and relational difficulties, risk of suicide and self-harm in adulthood [57]. Self-injurious behavior seriously threatens adolescent mental health; self-cutting is widespread among patients with BPD [58, 59], in both community and clinical settings. Self-harm is influenced by multiple factors, including social and interpersonal stressors, neurobiological background, emotional dysregulation, and ACEs [60]. ACEs increase the risk of attempted suicide 2-to 5-fold. Depressed mood, illicit drug and alcohol use influenced the relationship between ACEs and suicide attempts, suggesting an important mediation of these factors [61]. In our sample, despite self-harming actions were frequent, there were no suicide attempts.

Finally, there is growing evidence to support that trauma increases the risk for psychosis and affects severity and type of psychotic symptoms, and frequency of comorbid conditions, including depression and substance use [62]. Indeed, Liu et al., [63] support the hypothesis that ACEs are associated with positive psychotic symptoms. Individuals who reported at least one ACE had twice the risk of experiencing positive psychotic symptoms, compared to individuals with no exposure to ACEs [63]. We here found a higher rate of bipolar disorder and schizoaffective disorder in the preCUD-onset group, while schizophrenia missed the target by little (*p* = 0.071). The finding that BPD mimicked bipolar and schizoaffective disorders provides an affective hue to our results. Other studies found psychotic symptoms to be high in CUD populations, one focusing on substance-related exogenous psychosis finding ego-dystonic symptoms, which we did not investigate specifically, to constitute a significant part of both transient and persistent psychosis associated with CUD [64], another indicating the centrality of dissociative symptoms in CUD-associated psychosis [65]. It is possible that glutamatergic-cannabinoid interactions contribute to dissociation, as shown in both animals [66] and humans [67]. It is also interesting that in our sample of preCUD-onset patients there were more cases of hallucinogen use than in the NO group. Hallucinogens mainly act through 5-HT_2A_ receptors [68]; such receptors interact with the glutamatergic system [69] involving also the mediation of the GABAergic system [70], thus adding to the complexity of neurotransmitter imbalance occurring in SUD and psychiatric disorders. Such complexity should be further explored in humans and animals.

### Limitations

Our study has several limitations that should be acknowledged. First, we lacked a systematic assessment of preCUD-onset conditions. Second, the cross-sectional design of our study prevents us from establishing causality. Third, our sample size for each subgroup was relatively small. Furthermore, the retrospective assessment of past conditions of relatives and patients is subjected to recall bias. Also, the data collection about preCUD-onset condition has not been systematic. Despite these limitations, we hope that our real-world study will stimulate further research.

## Conclusions

Patients with preCUD-onset conditions exhibit earlier initiation of cannabis use, an earlier age at onset of psychopathology, and earlier hospitalization. Our results suggest a link between preCUD-onset conditions and disorders with a strong affective component, such as bipolar disorder, schizoaffective disorder, ADHD, and BPD. The use of cannabis among patients reporting a preCUD-onset condition may be considered a self-medication attempt to alleviate emotional or psychopathological impairments or traumatic experiences, and it may contribute to worse psychiatric outcomes.

## Data Availability

The first and corresponding authors will provide anonymized data upon reasonable request.
